# Glucuronoyl Esterase Screening and Characterization Assays Utilizing Commercially Available Benzyl Glucuronic Acid Ester

**DOI:** 10.3390/molecules201017807

**Published:** 2015-09-25

**Authors:** Hampus Sunner, Maria-Despoina Charavgi, Lisbeth Olsson, Evangelos Topakas, Paul Christakopoulos

**Affiliations:** 1 Industrial Biotechnology, Department of Biology and Biological Engineering, Chalmers University of Technology, Gothenburg SE-412 96, Sweden; E-Mails: hampus.sunner@chalmers.se (H.S.); lisbeth.olsson@chalmers.se (L.O.); 2 Wallenberg Wood Science Centre, Teknikringen 56-58, Stockholm SE-100 44, Sweden; 3 Biotechnology Laboratory, School of Chemical Engineering, National Technical University of Athens, 5 Iroon Polytechniou Str., Zografou Campus, Athens 15780, Greece; E-Mails: chem.marianna@gmail.com (M.-D.C.); vtopakas@chemeng.ntua.gr (E.T.); 4 Biochemical Process Engineering, Division of Chemical Engineering, Department of Civil, Environmental and Natural Resources Engineering, Luleå University of Technology, Luleå SE-971 87, Sweden

**Keywords:** glucuronic acid, glucuronoyl esterase, enzymatic assay, benzyl glucuronic acid ester, enzyme kinetics, Michaelis-Menten parameter estimation

## Abstract

Research on glucuronoyl esterases (GEs) has been hampered by the lack of enzyme assays based on easily obtainable substrates. While benzyl d-glucuronic acid ester (BnGlcA) is a commercially available substrate that can be used for GE assays, several considerations regarding substrate instability, limited solubility and low apparent affinities should be made. In this work we discuss the factors that are important when using BnGlcA for assaying GE activity and show how these can be applied when designing BnGlcA-based GE assays for different applications: a thin-layer chromatography assay for qualitative activity detection, a coupled-enzyme spectrophotometric assay that can be used for high-throughput screening or general activity determinations and a HPLC-based detection method allowing kinetic determinations. The three-level experimental procedure not merely facilitates routine, fast and simple biochemical characterizations but it can also give rise to the discovery of different GEs through an extensive screening of heterologous Genomic and Metagenomic expression libraries.

## 1. Introduction

Glucuronoyl esterases (GEs) are a recently discovered group of enzymes, which have been attributed the functional role of hydrolyzing the ester bonds between lignin alcohols and the 4-*O*-methyl-d-glucuronic acid side chains of xylan in plant cell walls [1,2]. These ester bonds constitute one of several bond types postulated to link lignin to carbohydrates (LC-bonds) in lignified plant matter [3]. As potential hydrolysers of this type of ester bonds, GEs may prove valuable as research tools for the investigation of lignin and LC-bond chemistry, as well as in a broad range of industrial applications for the biocatalytic processes of lignocellulose degradation and modification of lignocellulosic materials.

The first GE, *Sc*GE1 from *Schizophyllum commune*, was discovered and characterized in 2006 [1]. Its gene sequence was determined in 2007, leading to the discovery of the *Hypocrea jecorina* GE Cip2 [4] whose structure was determined in 2011 [5]. The *Sc*GE1 sequence initiated the establishment of carbohydrate esterase (CE) family 15 in the CAZy classification [6]. In addition, two GE enzymes from *Phanerochaete chrysosporium* (*Pc*GE1 and *Pc*GE2) [7], two from *Myceliophthora thermophila* (*St*GE1 [8] and *St*GE2 [9]), one from *Podospora anserina* (*Pa*GE1) [10] and one from *Cerrena unicolor* (*Cu*GE) [11] have been characterized. Structural determination of a catalytically inactive *St*GE2 mutant in complex with methyl 4-*O*-methyl-β-d-glucopyranuronate [12] provided novel insight into the structural determinants involved in substrate recognition and catalytic mechanism of GEs. To date, despite the new findings on CE15 family members, their physiological role and function remain to be fully delineated.

The discovery and characterization of GEs rests upon substrates that are products of laborious organic synthesis procedures, e.g., [13,14,15], thus limiting their accessibility and impeding the development and utilization of this promising class of enzymes. To broaden the scope of GE research, as well as to enhance its applicability in the sector of Industrial Biotechnology, standardized assays based on readily available substrates are required. Different types of assays have different requirements. For screening, simple and sensitive assays that are positive for a wide range of substrate specificities are ideal. To assess screening results, specificity is preferred over generality, whereas for enzyme purification purposes simplicity is preferable. Furthermore, for characterization, high precision and a large dynamic range are key features. Much of the value of characterization assays lies in activity comparisons between different enzymes using the same substrate. A kinetic GE assay on a substrate easily attainable for research purposes would therefore be the ideal.

The aim of this work is to present well-designed assay methods for the determination and screening of novel GE activity, using the commercially available benzyl d-glucuronic acid ester (BnGlcA) as a substrate. This substrate resembles the postulated β-*O*-4 α-carbon ester bond of d-glucuronic acid and constitutes a subset of the diaryl benzyl LC-ester model 1-(4-hydroxy-3-methoxyphenyl)-1-(α-d-glucuronate)-2-(2-methoxyphenoxy)-3-propanol that was recently synthesized and demonstrated to be hydrolysable by *St*GE2 [16]. We describe the application of this substrate in (a) a Thin Layer Chromatography (TLC) assay for qualitative activity assessment (b) a spectrophotometer-based assay useful for screening, and (c) an HPLC-based assay allowing more precise activity determinations, enabling enzyme kinetics characterization. In addition, TLC and spectrophotometric methods are less costly and time consuming, and there is no need for complicated instruments for the analysis.

## 2. Results and Discussion

### 2.1. Thin Layer Chromatography Assay

As a method for qualitative detection of GE activity, the TLC assay is a straightforward application of the *N*-(1-naphthyl)ethylenediamine dihydrochloride-based method [1,17], with the option to use an H_2_SO_4_-anisaldehyde reagent for development.

**Figure 1:**
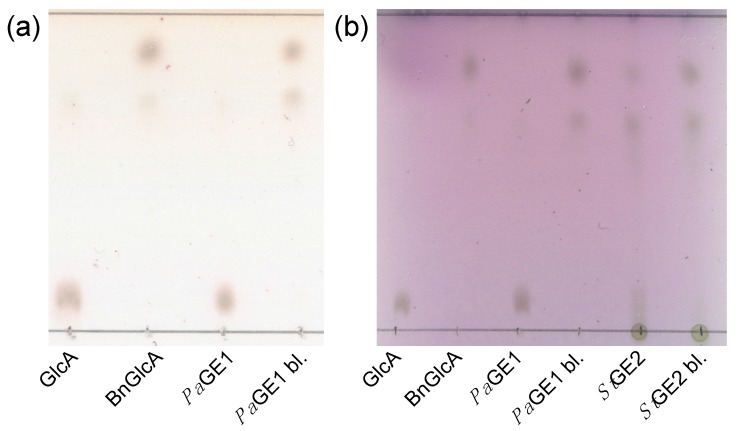
TLC chromatograms of two different plates. GlcA and BnGlcA were used as standards. As samples, BnGlcA that had been incubated with either of *St*GE2, boiled *St*GE2 (*St*GE2 bl.), *Pa*GE1 or boiled *Pa*GE1 (*Pa*GE1 bl.) was used. Plate (**a**) was developed using the *N*-(1-naphthyl)ethylenediamine dihydrochloride reagent and Plate (**b**) using the H_2_SO_4_-anisaldehyde reagent.

The TLC assay methodology was validated on purified *Pa*GE1 and concentrated *St*GE2 culture filtrate. Figure 1 presents the TLC chromatograms and demonstrates the plate appearances for both suggested developing reagents. The substrate spot in the *Pa*GE1 sample lanes have completely disappeared while the reduced intensity of corresponding spot in the crude *St*GE2 culture filtrate shows an incomplete enzymatic reaction. The analysis had a runtime of 30 min (4.7 cm rise) and for both reagents complete color development required 10 min at 100 °C. Color fading and storage corresponded to what has been previously reported [17]. GlcA and BnGlcA had *R*_f_s of 0.1 and 0.85, respectively. The TLC image shows a secondary spot of varying intensity in the BnGlcA substrate at *R*_f_ 0.7. This could be due to ring-closing isomers or dimers of the substrate and is consistent with the appearance of BnGlcA as a double peak in HPLC. The prominence of this spot in the culture filtrate samples indicates that the composition affects the equilibrium.

### 2.2. Spectrophotometric Assay

As a method for screening and activity estimation, the spectrophotometric assay uses NAD^+^-dependent oxidation of GlcA by uronate dehydrogenase (UDH) [18] as implemented in the uronic acid detection kit (K-URONIC Megazymes, Ireland) for quantification of the GlcA released by GE activity in a discontinuous coupled-assay protocol.

The methodology of the spectrophotometric assay was verified, and the detection limit determined, using concentrated *St*GE2 culture filtrate, chosen in order to resemble the conditions of a screening assay. Table 1 presents the detected activities for the culture filtrate in two separate dilution series, of four or ten times initial dilution. The lowest detected activity was 0.74 mU for the 9 µL sample (Table 1), corresponding to 0.9 µg *St*GE2. From these results, it can be concluded that the detection limit is at least 1 mU for a 1-tailed significance of p ~ 0.001. This corresponds to a detection limit of at least 4 mU/mL for an undiluted sample. Accepting a lower significance level, such as the customary *p* < 0.05, would give a better detection limit.

**Table 1:** Results from the spectrophotometric assay for an *St*GE2 culture filtrate for two separate dilution series. Sample volume in the first column refers to the volume of undiluted culture filtrate. Activity is calculated (*i*) separately for each dilution and (*ii*) for each series as a regression on all dilutions. For each sample, the determined activity (Det.Act.) and the volumetric activity (Vol.Act.; based on the undiluted sample) are presented. The coefficient of determination (r^2^) and the 1-tailed significance for each slope (p) are given. The analysis is also outlined in Figure 3.

Undil. Sample Vol. (µL)	Det.Act (mU)	Vol.Act. (mU/mL)	Slope (Au/mL/min)	r^2^	p 1-Tail
*StGE2 culture filtrate with an initial dilution of ten times in H_2_O*					
9	0.74	82.1	15.4	0.906	0.00095
13.5	1.14	84.2	15.8	0.977	1 × 10^−5^
18	1.60	88.7	16.6	0.988	2 × 10^−6^
*regression on all dilutions*		88.0	16.5	0.964	2 × 10^−9^
*StGE2 culture filtrate with an initial dilution of four times in H_2_O*					
22.5	1.64	72.9	13.7	0.987	3 × 10^−6^
45	3.53	78.4	14.7	0.997	2 × 10^−8^
67.5	5.12	75.8	14.2	0.999	1 × 10^−6^
*regression on all dilutions*		76.6	14.3	0.997	1 × 10^−16^

### 2.3. HPLC Assay and Kinetic Parameter Estimation

As a precision method for GE activity determination and kinetic parameter estimation, the HPLC method uses a stopped-reaction protocol followed by reverse-phase HPLC separation and quantification of the released benzyl alcohol by its UV absorbance at 254 nm.

**Figure 2:**
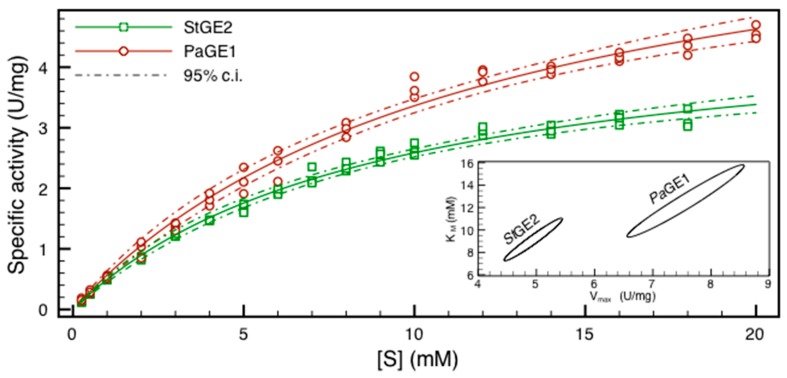
A least-squares regression fit of the Michaelis-Menten equation to the assay rate data for *Pa*GE1 and *St*GE2. The dashed lines in the main graph and the inset contour plot present two different views of the 95% confidence intervals of the parameter estimates.

The HPLC assay was validated by using it for the estimation of the kinetic parameters of purified *Pa*GE1 and *St*GE2. Table 2 presents the determined kinetic parameters and Figure 2 shows the measured specific activities and the fitted Michaelis-Menten functions with their 95% confidence intervals. When fitting the experimental data, both fits gave a correlation coefficient for *K*_m_ and *V*_max_ of 0.97. Because of this parameter dependence, which is to be expected when the maximum substrate concentration is not sufficiently high compared to *K*_m_, the 95% confidence intervals, shown as a contour plot in the inset graph of Figure 2, could not be computed from the standard errors and were instead constructed by a non-parametric method. To assess the limitations for the assay conditions, the half-life of BnGlcA was estimated at 30 °C and 40 °C and found to be 1.1 and 2.6 h at 40 °C and 3 and 6 h at 30 °C for pH 6.2 and 5.8, respectively.

**Table 2:** The kinetic parameters of *Pa*GE1 and *St*GE2 on BnGlcA as determined in the HPLC assay. The standard errors were calculated from the covariance matrix of the parameter fitting and the 95% confidence intervals were estimated as described in the Experimental Section.

	*K*_m_ (mM)		*V*_max_ (U/mg)		*K*_cat_ (s^−1^)	
	*K*_m_ (s.e.)	95% c.i.	*V*_max_ (s.e.)	95% c.i.	*K*_cat_ (s.e.)	95% c.i.
***Pa*GE1**	12.1 (0.8)	9.4–15.8	7.5 (0.2)	6.6–8.6	7.8 (0.3)	6.9–9.0
***St*GE2**	8.9 (0.5)	7.3–11.1	4.9 (0.1)	4.4–5.4	3.5 (0.1)	3.2–3.9

### 2.4. Comparison of the Three BnGlcA Assay Procedures

BnGlcA is easily used for a TLC-based assay for straightforward assaying without equipment requirements (Figure 1). This method constitutes a fast qualitative screening of sample activity, ideal for protein purification and useful for simple screening assays.

The results from the spectrophotometric assay indicate that BnGlcA can be used as substrate in assays to detect enzyme activities with a detection limit of <4 mU/mL, corresponding to 0.9 µg for *St*GE2. The applicability of the screening assay depends on how the aforementioned detection limit compares to the activity of actual titers attained in screening experiments. Literature titers for native expressions are 10 U/mL for *S. commune* [1] on methyl ester of 4-*O*-methyl GlcA, 19–120 mU/mL for *P. chrysosporium* [2] and 77 mU/mL for *M. thermophila* [8] both on 4-nitrophenyl 2-*O*-(methyl 4-*O*-methyl-α-d-glucopyranosyluronate)-β-d-xylopyranoside. No reliable data is available to directly estimate the corresponding activities on BnGlcA for these enzymes. However, for *Pa*GE1 the activity on 2 mM BnGlcA determined in this study (1 U/mg) is somewhat higher than the activity exhibited on 2 mM 4-nitrophenyl 2-*O*-(methyl 4-*O*-methyl-α-d-glucopyranosyluronate)-β-d-xylopyranoside (0.6 U/mg [10]), showing that for this enzyme, a direct comparison is possible. Furthermore, the calculated detection limit for *Pa*GE1 and *St*GE2 with the spectrophotometric assay is less than 1 µg/mL, which would be sufficient for detecting and quantifying the GE titers that have been previously reported for native GE expression (10 µg/mL [1] and 5 µg/mL [8]). The determining factor for the applicability of BnGlcA in screening assays is consequently the specific activity that each target enzyme exhibits for this substrate.

The spectrophotometric assay is, in the microplate format, also applicable for activity determination in expression libraries or during a downstream process, such as protein purification. As the suggested protocol is designed to fulfill several requirements at once—statistical significance, low detection limit and quantification over two orders of magnitude—it can be simplified to match the assay requirements. However, appropriate reference samples should be used and the limited linear range of the assay should be considered. For simple activity detection TLC analysis using BnGlcA may also be a suitable option.

The measured half-times around pH 6 illustrate the pH and temperature dependence of the BnGlcA background hydrolysis and show that the assay is practically limited to pH 4–6 and to temperatures below 50 °C. Even at moderate pH and temperature, background hydrolysis may cause activity underestimation if not taken into account, and at high pH and temperature assay accuracy cannot be maintained. Hence, BnGlcA is not reliable at neutral to basic pH or at high temperatures. However, as GEs constitute a class of enzymes where substrate specificities have not yet been thoroughly explored, the usable range of conditions would be applicable for most assay situations.

The HPLC-based assay has a broader applicability than the spectrophotometric assay. The ability to stop the enzymatic and background reactions allows the assay to be applied over a wider range of pH and temperature. The spectrophotometric assay is not suitable for kinetics, as the reaction system in that assay is too complex to accurately estimate initial enzymatic hydrolysis rates. In contrast, the HPLC assay uses a simpler reaction system where the enzymatic and background reactions are stopped after a certain incubation time. This, in conjunction with the use of one boiled-enzyme control for each sample, gives the HPLC assay the precision and accuracy required for kinetic characterization.

The apparent factors that decide the applicability of BnGlcA for the determination of kinetic parameters are the solubility of the substrate, the affinities of the target enzymes and the pH and temperature limits of the assay. The hydrophobic BnGlcA has a limited solubility without co-solvents or emulsifiers, and their usage may have appreciable effects on reaction kinetics. Determining a *K*_m_ parameter requires a substrate concentration of at least double the *K*_m_ value. Thus, uncertainty increases with an increasing *K*_m_ and the high *K*_m_s observed for several enzymes on BnGlcA (see Table 2 and [11]) should therefore be interpreted with caution. In addition, to the authors’ knowledge, there is no report on the significance of *K*_m_ values determined with soluble substrates as estimators of the affinity or reaction rate on insoluble ones. Regardless of their validity, the kinetic parameters estimates can be used practically for activity prediction inside of the assayed substrate range, although predictions based on the measured activities rather than derived kinetic parameters may give more accurate results.

The third factor, which is the limited range of usable assay conditions, is not necessarily a drawback for activity comparison. The ability to assay all available GEs and determine the kinetic properties at a single pH and temperature, even if not the optimal for the individual enzyme, would nonetheless provide a common denominator and to this end a pH of 6 at temperatures of 30–40 °C is a good starting point.

Our work outlines strategies and suggests methods that can be used to overcome the pitfalls that could otherwise be encountered when designing, applying, reporting on and interpreting BnGlcA-based assays, such as those related to the instability and limited solubility of BnGlcA. BnGlcA is indeed a suitable candidate substrate to be routinely employed for general activity determinations, as well as for biochemical characterization of GEs.

## 3. Experimental Section

### 3.1. Chemicals and Enzymes

BnGlcA was purchased from Carbosynth (Compton, UK). *St*GE2 and *Pa*GE1 were produced and purified as described previously [9,10], while *St*GE2 was used before purification as a concentrated StGE2 culture filtrate in the TLC and spectrophotometric assays. The *P. anserina* strain used for the expression of *Pa*GE1 in the present work was kindly provided by Jean-Guy Berrin (INRA, Aix Marseille Université, Marseille, France). Deionized H_2_O was used for all experimentation. All other chemicals were of HPLC grade and purchased from Sigma-Aldrich (St. Louis, MO, USA).

### 3.2. Thin-Layer Chromatography Assay

10 µL of 5 mM BnGlcA in 50 mM pH 6 acetate buffer was incubated for 30 min by purified *Pa*GE1 (50 µg; 35 °C) or *St*GE2 culture filtrate (1 µg; 45 °C). 0.5 µL samples were blotted onto Merck silica gel 60 F_254_-precoated plates and analysed, as previously described [1]. H_2_SO_4_-anisaldehyde reagent (170 mL methanol, 20 mL glacial acetic acid, 10 mL H_2_SO_4_ and 1 mL 4-anisaldehyde) or *N*-(1-naphthyl)ethylenediamine dihydrochloride reagent [17] were used as developing reagents.

### 3.3. Spectrophotometric Assay

The spectrophotometric assay uses the K-URONIC kit (Megazyme, Bray, Ireland) and is based on the oxidation of d-glucuronic acid to d-glucarate by uronate dehydrogenase which is monitored by measuring the absorbance of NADH at 340 nm [18], a wavelength where benzyl alcohol and BnGlcA have negligible absorbance. Measurements were performed with 250 µL well volume in a 96-well flat-bottomed microplate (Sarstedt 82.1581, Nümbrecht, Germany) in absorbance mode (FLUOstar Omega, BMG Labtech, Ortenberg, Germany). BnGlcA was dissolved in 100% dimethyl sulphoxide (DMSO) to a 100 mM substrate stock and stored at −20 °C. To determine effective molar absorptivity and plate reader linear range, a standard curve of GlcA covering the entire detection range was generated. For each subsequent analysis, at least one upper-range standard was included as an external standard.

**Figure 3:**
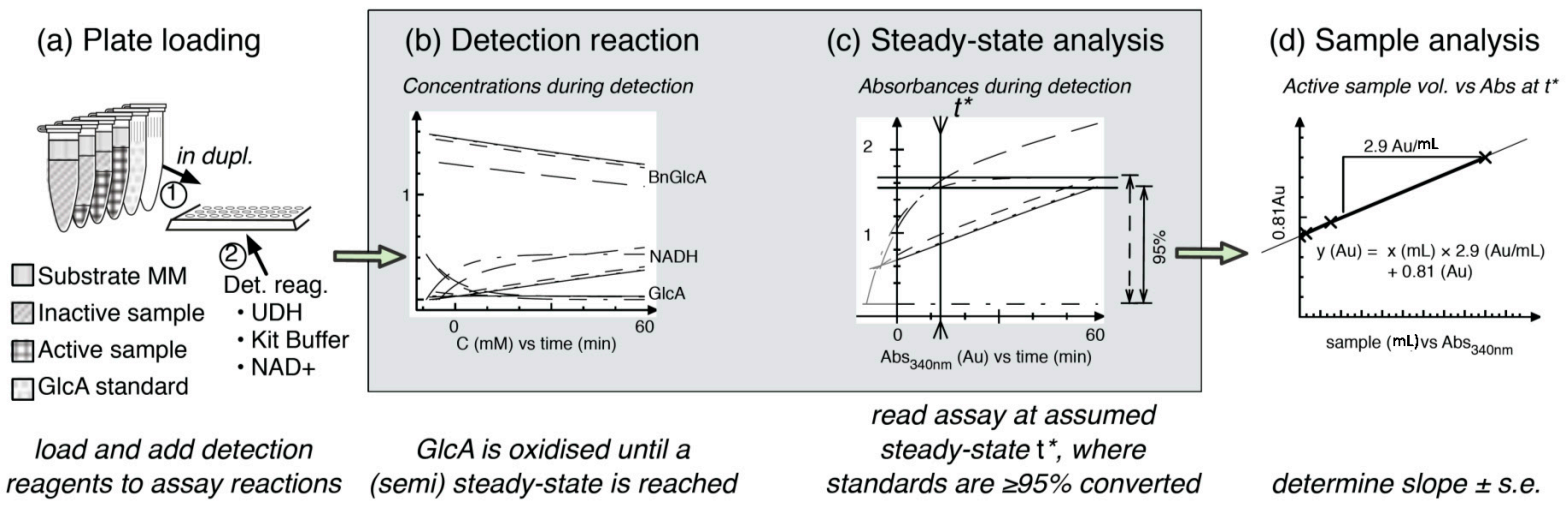
Summary of the spectrophotometric assay. After the enzymatic reaction the samples are; (**a**) loaded together with controls and standards into a 96-well microplate and supplemented with detection reagents; In the detection reaction (**b**) GlcA released during the enzymatic reaction is oxidized while BnGlcA is continuously hydrolyzing to release additional GlcA. A (semi) steady-state is eventually reached at time *t*; (**c**) The detection reaction is assumed to be at steady-state when the included standard(s) have reached 95% conversion (time t*); (**d**) The slope of sample amount *vs.* absorbance at t* is calculated.

The *St*GE2 culture filtrate was diluted in H_2_O and a portion of the dilution was heat-inactivated whereafter active and boiled culture filtrate were recombined at varying proportions to create a dilution series with the same protein concentration and background composition but with varying activity. Each dilution was assayed in duplicate in 2 mM BnGlcA at 45 °C for 30 min. The samples were transferred to a microplate in technical duplicates and supplemented with 50 µL of cold detection solution (Figure 3a), consisting of 40% buffer, 40% NAD^+^ and 4% UDH, as included in the K-URONIC kit. The detection reaction was followed by the NADH formation by taking the absorbance every 30–60 s for 60 min. The GlcA released during the enzyme reaction is quickly hydrolysed and a semi steady-state condition is eventually reached (Figure 3b), when the GlcA concentration is slowly decreasing, following the decreasing concentration of BnGlcA, which is continuously hydrolysed during the detection reaction.

The steady-state condition was estimated by analysis of the included standards: samples were assumed to be in a steady-state from the first time point when all the included standards had reached 95% of their plateau values (Figure 3c). A linear regression was made for the absorbance values at this time point as a function of the amount of active enzyme in each sample of the dilution series (Figure 3d). A significance value for the sample being positive could then be obtained from the *t-*statistic of the slope of this line. In order to estimate the sample activity, the slope and its standard error were converted to specific activities by the specific absorbance of the NADH as estimated by the standard curve.

### 3.4. HPLC-Based Assay

BnGlcA was dissolved in 100% DMSO to stock solutions of 100 mM and 250 mM, which were aliquoted and stored at −20 °C. Before each experiment, the substrate was diluted in a 100 mM pH 6 phosphate buffer into 15 concentrations 0.25–20 mM (*Pa*GE1) or 16 concentrations 0.25–18 mM (*St*GE2). For each sample, 470 µL of the substrate solution were supplemented by 30 µL *Pa*GE1 (7.1 µg) or *St*GE2 (18 µg) in 20 mM pH 8 Tris-HCl buffer and incubated at 35 °C and 45 °C respectively. For each sample, a corresponding boiled-enzyme control was made. After 30 min, 100 μL of glacial acetic acid were added to stop the reaction and each sample was analysed by HPLC in duplicate at 20 °C overnight. Separation of BnGlcA and benzyl alcohol was achieved in a C18 Nucleosil column (250 mm × 4.6 mm) (Macherey Nagel, Düren, Germany) using acetonitrile/water (6:4 *v*/*v*) with a flow rate of 0.4 mL·min^−1^ (Jasco PU 987, Tokyo, Japan) at room temperature. The benzyl group was detected by UV absorption at 254 nm (UV-Vis ProStar 335 Diode Array Detector, Agilent Technologies). For estimation of the BnGlcA background hydrolysis, the hydrolysis rates of 1 mM BnGlcA in 50 mM acetate-phosphate buffers at pH 5.8 and 6.2 (as measured at 40 and 30 °C) were monitored by HPLC at minimally 6 time points per sample over at least 30 min.

*K*_m_ and *V*_max_ were estimated by a non-linear least squares optimization of the Michaelis-Menten equation. This was done using the lmfit (lmfit.github.io/lmfit-py) python (python.org) package. lmfit also provided non-parametric estimates of the two-dimensional confidence intervals by *F*-testing the χ^2^s of the optimal fit to each alternative *K*_m_ and *V*_max_ combination close to the best fit. The confidence intervals were then extracted from the resulting probability matrix, which was also used to generate a contour plot of each fit (combined for both enzymes in Figure 2).

## 4. Conclusions

The lack of available substrates for GE assays has been hampering GE research and a new reference substrate is required. The substrate should be universally available, cheap and easily applicable. BnGlcA is a low-cost, commercially available substrate and our work shows that it can be used for assaying GE activity in multiple contexts. It can be exploited non-quantitatively in a TLC-based assay that is fast to apply, scales easily and does not require any special equipment. With the use of a spectrophotometer, a quantitative coupled-enzyme assay can be set up. The presented protocol allows quantification of GE activity as well as screening, with a detection limit below 1 mU or 12 µg, as estimated for the two GEs studied. An HPLC-based GE assay on BnGlcA, using a standard C18 column for separation and UV absorbance to monitor the release of benzyl alcohol, allows determination of activity with higher precision and accuracy in a larger dynamic range augmenting the methodology’s potential for enzyme characterization. In addition, the spectrophotometric GE assay presented in this paper, is suitable for the high-throughput analysis of screening expression libraries that will enable the discovery of novel GEs. BnGlcA, a low cost and commercially available substrate, can be used in laboratory practice for a simple and fast assay approach using three different analytical methodologies supporting the discovery of novel GEs from saprophytic microorganisms.

## Supplementary Materials

**suppl_microplate _protocol.pdf:** Detailed protocol for the microplate assay;

**suppl_data_analysis.xlsx:** Template for statistical hypothesis testing of microplate assay results;

Supplementary materials can be accessed at: http://www.mdpi.com/1420-3049/20/10/17807/s1.
