# Supplementary material for: Glucuronoyl Esterase Screening and Characterization Assays Utilizing Commercially Available Benzyl Glucuronic Acid Ester
Source: Molecules. 2015 Sep 25;20(10):17807–17. doi: 10.3390/molecules201017807 (PMC6332307; doi:10.3390/molecules201017807)
Supplement: Supplementary file 1 [file molecules-20-17807-s001.zip › supplementary/suppl_microplate_protocol.pdf]

BnGlcA UDH-Coupled GE Screening Assay

Exemplified by Application on Two Culture Filtrates

A microplate-based enzyme assay for glucuronic acid esterase activity using benzyl glucuronic acid ester as substrate and the K-URONIC kit (Megazyme) for coupled-assay detection.

Contents

1. Method Description S1
2. Equipment and Reagents S2
3. Sample List and Microplate Layout S2
4. Preparations S2
5. Procedure S5
6. Data Analysis S6
7. Protocol Notes and Variations S8

1. Method Description

This protocol describes a typical application of a more general protocol, as applied on two culture filtrates, E and F, in which we want to screen for, and if possible quantify, GE activity.

As presented here, this protocol is heavily optimized for screening for low GE activities (<100 mU/mL), with activity estimation as a secondary objective. See §7 for suggestions for optimizations and for adaptations for other use cases.

This protocol outlines the steps required for the estimation of sample activity and a supplementary spreadsheet (suppl_data_analysis.xlsx) shows how to compute the statistical significance for each positive sample.

1.1. Method Details

The assay is based on the commercially available substrate benzyl glucuronic acid ester (BnGlcA) and uses a discontinuous coupled-assay approach with an initial enzyme reaction in vials followed by a detection reaction in a microplate. For detection, a glucuronic acid (GlcA) quantification kit (K-URONIC; Megazyme, Bray, Ireland) is used. The detection is based on the quantification of NADH that is generated from the oxidation of liberated glucuronic acid to D-glucarate by uronate dehydrogenase.

- The enzyme reaction takes place at pH 6 in 25 mM PO_4 buffer and the detection reaction around pH 8 in the kit buffer
- Each culture filtrate is analysed in a dilution series, increasing the effective linear range of the assay and giving better statistic support for positive/negative discrimination.
- As BnGlcA has a significant pH- and buffer-dependant background hydrolysis around pH 6, boiled culture filtrate is used as diluent for the dilution series in order to have the same background in all samples.
- The volumes and concentrations used in this protocol application are optimised for a minimal detection limit and adapted for technical duplicates
- The reaction buffer (25 mM PO_4) is optimised for minimal interference during the detection phase. The presence of other buffers should be minimized.

- This application has an expected detection range of at least 5–500 mU/mL, though linearity has not been assessed.
- For high-activity samples, the assay reliability can be increased by: decreasing the enzyme reaction incubation time (~10 min) minimizing interfering species by purifying or buffer-exchanging the samples, or by increasing the pre-dilution factor.
- Deionized water is used for all dilutions and solutions are kept on ice unless noted

2. Equipment and Reagents

- Microplate photometer, usable at 340 nm for absorbance measurements
- Absorbance plates suited for 340 nm and 250 μL volume
- Heating block capable of >100 °C for enzyme inactivation
- Heating block for >0.5 mL tubes, ideally mixing, for reaction incubation
- Megazyme K-URONIC uronic acid quantification kit
- 100 mM PO_4 buffer with a target pH of 6.0 at 25 mM and 30 °C
- 2–5 mM Glucuronic acid in H_2O or pH 6 PO_4 buffer (GLCA STANDARDS STOCK)
- 100 mM Benzyl glucuronic acid ester (BnGlcA) stock in water-free DMSO

3. Sample List and Microplate Layout

	1	2	3	4	5	6
A	S0	H_2O	S0	H_2O	F0A	F0A
B	S1	BB	S1	BB	F1A	F1A
C	S2	SB	S2	SB	F2A	F2A
D	S3	ISC	S3	ISC	F3A	F3A
E	E0A	E0A	E0B	E0B	F0B	F0B
F	E1A	E1A	E1B	E1B	F1B	F1B
G	E2A	E2A	E2B	E2B	F2B	F2B
H	E3A	E3A	E3B	E3B	F3B	F3B

The table above show a suggested plate layout of the following samples, each of 450 μL volume and loaded onto the plate as technical duplicates of 200 μL each

- S0/BB — BUFFER BLANK—25 mM PO_4 pH 6.0 blank
- S1…S3 — Selected GLCA STANDARDS (60 μM, 180 μM, 540 μM)
- H_2O — Water blank
- SC — SUBSTRATE CONTROL
- ISC — INCUBATED SUBSTRATE CONTROL
- E,F0...3A,B — REACTION SAMPLES of E and F
- four culture filtrate dilutions from 0 (ENZYME CONTROL) to 4 (270 μL active sample)
- each in two reaction duplicates: A and B

4. Preparations

Prepare the K-URONIC kit according to kit instructions.

4.1. PO_4 Buffer Dilutions

Dilute from a common stock so that all buffers will have the same pH after final dilution to 20 mM in the detection reaction.

Prepare the following PO_4 buffers from 100 mM PO_4 buffer stock (target pH of 6.0 at 25 mM and 30 °C)

- 100 mM *for preparation of standards*
- 65.8 mM *for BNGLCA REACTION SOLUTION*
- 25.0 mM *for BUFFER BLANK and for the blank in the GlcA standard curve*

4.2. GlcA Standards and Standard Curve

Prepare and freeze GlcA standards. The standard curve needs to be made once, but at least one standard should be included in every assay run. Ideally the same standard preparation should be frozen in aliquots and used every time.

- Make a dilution series of GLCA STANDARDS STOCK in pH 6 PO_4 for a final buffer concentration of 25 mM
- *The expected linear range is within 30 µM–1 mM*
- *Suggested concentrations: 60, 180, 360, 540, 720 µM in 450 µL aliquots (stored at −20 °C)*
- Aliquot the dilution series (GLCA STANDARDS)
- Load 200 µL of each GLCA STANDARD into a microplate in (at least) technical duplicates using 25 mM PO_4 buffer for the blanks
- Prepare DETECTION SOLUTION and add 50 µL to each well
- Measure the absorbance at 340 nm every 30 s–60 s for 30–60 min
- *Ideally at a defined temperature 20–30 °C*
- From the measurements, determine the plate reader linear range and the effective molar extinction coefficient (ε)

4.3. Diluted Enzyme Samples

As culture filtrates often have background absorbance at 340 nm, the culture filtrates may need to be pre-diluted so that the final absorbance during detection does not end outside the plate reader linear range.

For each culture filtrate to be screened:

- Estimate the background absorbance at 340 nm as it would be read in the microplate reader with 250 µL well volume.
- Dilute the culture filtrate in H_2O so that the background absorbance of the resulting DILUTED ENZYME SAMPLE is less than 2 Au and so that the expected volumetric activity (if estimable) is within the detection range (ideally ~0.1 mU/mL). (This is the PRE-DILUTION FACTOR)
- *The diluent should be H_2O rather than PO_4 to minimise buffer interference during detection*

For the samples defined in §3, 2.4 mL of each DILUTED ENZYME SAMPLE is required

4.4. BnGlcA Reaction Solution

To minimize background hydrolysis, BnGlcA Reaction solution should be prepared freshly just minutes before each enzyme reaction and kept on ice.

100 mM BnGlcA is diluted in 65.8 mM PO_4 in the proportions 9:171.

180 µL *BnGlcA Reaction solution* is required for each Reaction sample and each Substrate blank (SC & ISC).

For the samples defined in §3, 180 µL of 100 mM BnGlcA in DMSO is diluted in 3420 µL of 65.8 PO_4.

4.5. Detection Solution

Detection solution should be finalized by adding NAD^+ and UDH (K-URONIC Bottle 3) just before usage, but kit buffer (Bottle 1) can be mixed with H_2O earlier the same day.

The Detection solution is comprised of an H_2O dilution of K-Uronic kit components:

- 40% of buffer (Bottle 1)
- 40% of NAD^+ (aliquots of Bottle 2)
- 4% UDH (Bottle 3)

50 µL Detection solution is required for each well on the plate

For the samples defined in §3, 2.6 mL Detection solution should be prepared

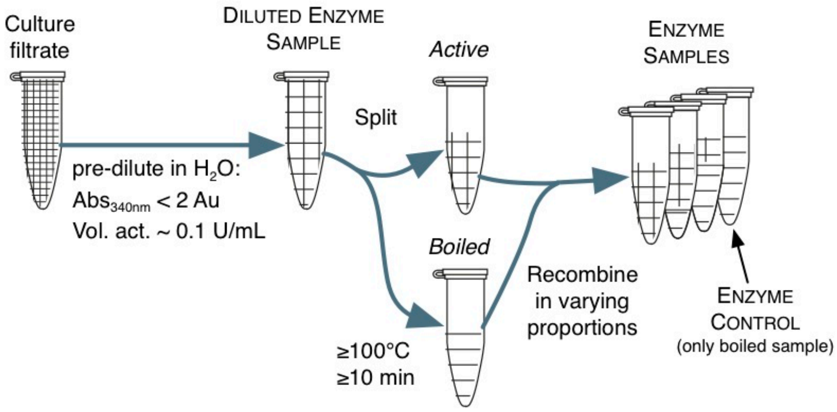

Figure S1. Each culture filtrate is diluted in H_2O into a Diluted Enzyme sample. A part of this is inactivated through boiling, and the active and boiled samples are recombined to a series of Enzyme Samples of varying activity with the same background (matrix).

4.6. Enzyme Samples

See Figure S1 for an overview of how the Enzyme Samples are prepared.

For each sample "X":

- Transfer 1670 µL to new vials (Boiled Diluted Enzyme sample)
- Boil the Boiled Diluted Enzyme samples: 30 min at ~110 °C is advisable for heat-tolerant enzymes.

- Create an ENZYME SAMPLES dilution series by preparing, in duplicates A and B:
- X0: 270 µL BOILED DILUTED ENZYME SAMPLE (X0 is an ENZYME CONTROL)
- X1: 261 µL BOILED DILUTED ENZYME SAMPLE + 9 µL DILUTED ENZYME SAMPLE
- X2: 220 µL BOILED DILUTED ENZYME SAMPLE + 50 µL DILUTED ENZYME SAMPLE
- X3: 270 µL DILUTED ENZYME SAMPLE

X0A and X0B are the replicates of Enzyme Sample X0.

The ACTIVE SAMPLE AMOUNTS (used for activity calculation) for the samples X1–X3 for this dilution scheme are

$$\textit{ACTIVE SAMPLE AMOUNTS} = \text{PRE-DILUTION FACTOR} \times \{0.009, 0.050, 0.270\}\ (\text{mL})$$

4.7. Substrate Controls

SUBSTRATE CONTROL is a control for the amount of substrate hydrolysed before incubation. INCUBATED SUBSTRATE CONTROL is a control for the amount of substrate hydrolysed during incubation

Prepare SUBSTRATE CONTROLS by adding 270 µL H_2O to two vials, one for SUBSTRATE CONTROL and one for INCUBATED SUBSTRATE CONTROL

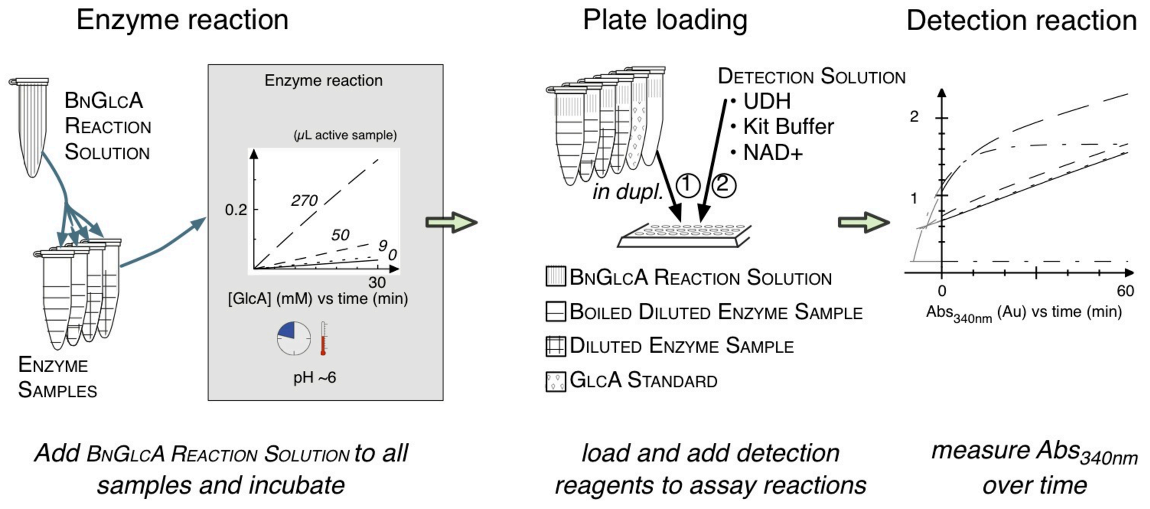

Figure S2. Schematic of the procedure for the enzyme and detection reactions.

5. Procedure

The main assay procedure—the enzyme and the detection reactions—is outlined in Figure S2.

5.1. Enzyme Reaction

For long incubations (e.g., 30 min), pre-heating the Enzyme samples before addition of BNGLCA REACTION SOLUTION is not required, though it would be recommended for shorter incubations (e.g., 10 min).

- Add 180 µL of BNGLCA REACTION SOLUTION to each ENZYME SAMPLE and to INCUBATED SUBSTRATE CONTROL (ISC)

- Incubate for 30 min at 30 °C, followed by cooling at +4 °C
- Also add 180 µL of BnGlcA Reaction solution to the Substrate Control (SC) and keep on ice

5.2. Plate Loading

Load each well with 200 µL of the appropriate sample, control or blank. Each GlcA standard, Enzyme Samples, *and* Substrate Control *of 450 µL each can thus be analysed in two technical replicates. Plate loading can be started while the enzyme reaction is incubating or once it is finished and cooling. Note that the enzyme reaction samples should be analysed* immediately *after the incubation.*

- Load standards (S0–S3), blanks (H_2O and BB) and Substrate Control (SC) into the microplate
- Finalize the Detection Solution: add NAD^+ and UDH
- Once cold, load the Enzyme Samples and the Incubated Substrate Control onto the plate
- Add 50 µL Detection Solution to each well
- Measure the absorbance at 340 nm every 30 s–60 s for 30–60 min

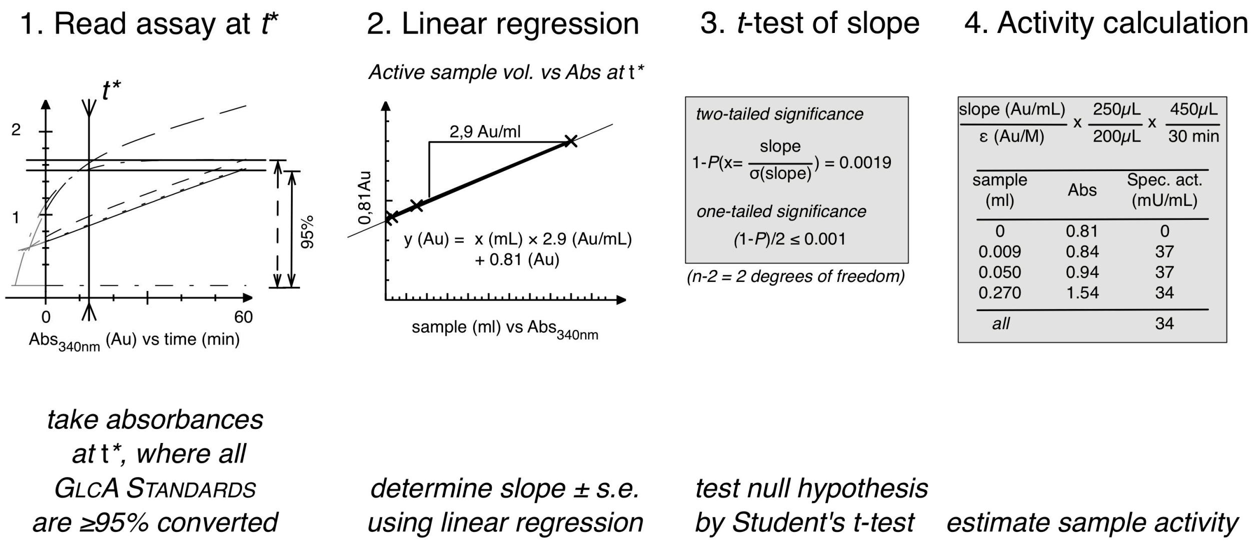

Figure S3. Schematic over the data analysis procedure. Steps 2 and 3 can be done by the associated spreadsheet.

6. Data Analysis

The data analysis is comprised of the following steps (outlined in Figure S3). The associated spreadsheet (suppl_data_analysis.xlsx) contains example data and can be used for the calculations where indicated:

1. Determine at what time point to read the assay
   - The absorbance values for all samples at a single time point (t*) should be chosen for analysis.
   - Because the BnGlcA hydrolyses continuously at the detection pH, the Enzyme Samples will not reach an absorbance plateau. Instead a steady-state, when the rate of GlcA oxidation is more or less constant, is reached.

- The steady-state is not trivial to detect, so t^* is instead chosen on the basis of the plateaus of the included GLCA STANDARDS.
- We arbitrarily decide that t^* is the time point when all included GlcA standards have reached 95% conversion
- The raw absorbance values for all ENZYME SAMPLES at t^* are used for the continued analysis
- Practical procedure:
  1. For each GLCA STANDARD, establish the average absorbance value of the completed reaction (for example the average absorbance at 30–60 min)
  2. For each GLCA STANDARD *SX*, determine the threshold absorbance when the reaction is at least 95% completed:

$$Abs_{SX,critical} \geq 95\% \times \left(Abs_{SX,plateau} - \mathrm{Abs}_{S0}\right) + \mathrm{Abs}_{S0}$$

  3. Determine the time t^*, when all GLCA STANDARDS has reached their 95% completion threshold.

2. Linear regression for each culture filtrate
   - For each screened culture filtrate, a linear regression of the ACTIVE SAMPLE AMOUNT vs Abs_{t^*} is made
   - The raw absorbance values for all ENZYME SAMPLES (including ENZYME CONTROL X0) at t^* are used
   - Practical procedure (done separately for each culture filtrate):
   - Tabulate all absorbance measurements at time t^* against the ACTIVE SAMPLE AMOUNT
   - Do a linear regression of the Absorbances vs ACTIVE SAMPLE AMOUNT
   - *The associated spreadsheet can be used for the regression, or serve as a template for it*
   - *For samples where the protein amount is known, it is more practical to base the regression on Specific Activity rather than Volumetric Activity.*

3. *t*-test of slope
   - A one-tailed significance of the slope can be calculated by Student's *t*-distribution if the standard error of the slope was calculated in the previous step.
   - *The associated spreadsheet will provide the significance, if used for the linear regression*

4. Activity calculation
   - The activity of each sample is calculated from the slope of the previous regression and the effective molar extinction coefficient (ε)

$$\mathrm{Activity} = \frac{slope}{\varepsilon} \times \frac{250\ \mu\mathrm{L}}{200\ \mu\mathrm{L}} \times \frac{450\ \mu\mathrm{L}}{30\ \mathrm{min}}$$

   - If the slope is given in Au/mL, the resulting activity is Volumetric Activity; If Au/mg, the resulting activity will be Specific Activity.

7. Protocol Notes and Variations

7.1. Protocol Adaptations

As written, the protocol is applicable both to screening and to activity estimation and for culture filtrates as well as for purified enzyme samples. **For screening** without activity estimation, a single dilution in three or more replicates would be superior. **For activity estimation**, reaction monoplicates with a wider and denser dilution series would be more suitable if the expected activity is not known.

7.2. Time and Volume Factors of Detection Reagent Addition

The hydrolysis rate of BnGlcA is much higher at pH 8 than at pH 6. Thus, two factors play a significant role for the rate of background hydrolysis in the detection reaction (and thus for assay precision):

1. Variations in the volume of DETECTION REAGENT that is added, causing small pH differences.
2. The time difference of the addition of DETECTION REAGENT for different samples (the start time offset).

The first point can be remedied by careful pipetting, using a pipetting robot or the injection syringe of the spectrophotometer.

The second point is handled the simplest by minimizing the error, and not correcting for it, by adding DETECTION REAGENT in a thought-through order, and by limiting the number of samples per batch.

Correcting for the time offset of each sample is possible, but impractical in a spreadsheet-based analysis. If the well-to-well read interval is the same as the pipetting interval, and the order is the same (*i.e.*, if a plate reader injection syringe is used), the plate reads will implicitly be offset-adjusted.

Adding both reagent solutions (BNGLCA REACTION REAGENT and DETECTION REAGENT) by plate reader-syringe and doing both reactions in the plate reader would be another way to enable easy offset correction, and may in itself be a way to increase precision and accuracy further.

7.3. Single vs. Multiple Time Point Analysis

For data analysis, only the absorbance from a single time point (or possibly the average of a very short interval) should be used from each sample: As the variation of absorbance of the ENZYME SAMPLES over time is not random, the analysis of multiple time points require using a "general linear regression" model in which the time can be factored into the equation. Although the common spreadsheet function "LINEST" is capable of this analysis, it is impractical to do in a spreadsheet, especially as the start time offset of each sample should be considered in this case.

7.4. The Effect of Sample Dilution and Reaction Length on Assay Accuracy

The higher activity and/or longer enzyme reaction incubation, the more the measured activity deviates from the "initial reaction velocity" Hence, more dilute samples give more credible activities and therefore discarding high-activity samples when calculating slopes should be considered. Also, the pH variation caused by the sample background itself is less for more dilute samples. Estimated activities should be considered with these factors in mind.

As BnGlcA hydrolyses during the enzyme reaction at pH 6, shorter enzyme reactions also increase accuracy, but require samples with more inherent activity.

7.5. One vs. Two-Tailed Significance

The significance test is made using a cumulative Student's *t*-distribution (spreadsheet function TDIST or T.DIST). The null hypothesis for the test is for the slope being zero. As a negative slope is a negative result (no sample activity), a 1-tailed distribution should be used to discriminate positive samples from negative ones. The 1-tailed probability (T.DIST.RT) can thus be interpreted as: the probability of the measured data on the predicate that the slope is zero or negative. A practical interpretation is that the chosen probability cut-off (e.g., $p < 0.05$) is an estimate of the fraction of false positives.
